# Rationale and design of a multicenter Chronic Kidney Disease (CKD) and at-risk for CKD electronic health records-based registry: CURE-CKD

**DOI:** 10.1186/s12882-019-1558-9

**Published:** 2019-11-20

**Authors:** Keith C. Norris, O. Kenrik Duru, Radica Z. Alicic, Kenn B. Daratha, Susanne B. Nicholas, Sterling M. McPherson, Douglas S. Bell, Jenny I. Shen, Cami R. Jones, Tannaz Moin, Amy D. Waterman, Joshua J. Neumiller, Roberto B. Vargas, Alex A. T. Bui, Carol M. Mangione, Katherine R. Tuttle, on behalf of the CURE-CKD investigators, Karen Agnew, Celestina Barbosa-Leiker, Ann Cooper, Mark Gargett, Marianne Zachariah, Peter Kim, Celestina Barbosa-Leiker, Carol Miceli, Robert W. Follett, Theona Tacorda, Susan Ettner, David Elashoff

**Affiliations:** 1 David Geffen School of Medicine at University of California, Los Angeles, CA 90095 USA; 2 UCLA Department of Medicine, Division of General Internal Medicine, 1100 Glendon Ave. Suite 900, Los Angeles, CA 90024 USA; 3 Providence St. Joseph Health, Providence Medical Research Center, Spokane, Washington USA; 4 University of Washington School of Medicine, Seattle, Washington USA; 5 Washington State University Elson S. Floyd College of Medicine, Spokane, Washington USA; 6 Los Angeles Biomedical Research Institute at Harbor-UCLA Medical Center, Torrance, CA USA; 7 VA Greater Los Angeles, Los Angeles, USA; 8 Washington State University College of Pharmacy and Pharmaceutical Sciences, Spokane, USA; 9 Charles R. Drew University of Medicine and Science, Los Angeles, USA; 10 RAND Corporation, Santa Monica, CA USA

**Keywords:** Chronic kidney disease, Electronic health records, Healthcare systems, Hypertension, Diabetes, Pre-diabetes, Registry, Study design

## Abstract

**Background:**

Chronic kidney disease (CKD) is a global public health problem, exhibiting sharp increases in incidence, prevalence, and attributable morbidity and mortality. There is a critical need to better understand the demographics, clinical characteristics, and key risk factors for CKD; and to develop platforms for testing novel interventions to improve modifiable risk factors, particularly for the CKD patients with a rapid decline in kidney function.

**Methods:**

We describe a novel collaboration between two large healthcare systems (Providence St. Joseph Health and University of California, Los Angeles Health) supported by leadership from both institutions, which was created to develop harmonized cohorts of patients with CKD or those at increased risk for CKD (hypertension/HTN, diabetes/DM, pre-diabetes) from electronic health record data.

**Results:**

The combined repository of candidate records included more than 3.3 million patients with at least a single qualifying measure for CKD and/or at-risk for CKD. The CURE-CKD registry includes over 2.6 million patients with and/or at-risk for CKD identified by stricter guide-line based criteria using a combination of administrative encounter codes, physical examinations, laboratory values and medication use. Notably, data based on race/ethnicity and geography in part, will enable robust analyses to study traditionally disadvantaged or marginalized patients not typically included in clinical trials.

**Discussion:**

CURE-CKD project is a unique multidisciplinary collaboration between nephrologists, endocrinologists, primary care physicians with health services research skills, health economists, and those with expertise in statistics, bio-informatics and machine learning. The CURE-CKD registry uses curated observations from real-world settings across two large healthcare systems and has great potential to provide important contributions for healthcare and for improving clinical outcomes in patients with and at-risk for CKD.

## Background

Chronic kidney disease (CKD) is a major public health problem affecting an estimated 30 million United States (US) adults and is the 9th leading cause of death in the US [1]. Patients with CKD suffer from high rates of premature morbidity including cardiovascular diseases and progression to end stage kidney disease (ESKD) as well as premature mortality [1]. In addition, CKD imposes a high financial burden accounting for over 7% of Medicare spending on ESKD patients per year, while less than 1% of the Medicare population are ESKD patients [2]. Thus, the care of CKD patients is a national legislative priority [3–5]. Despite several strategies to improve CKD prevention, early intervention and outcomes, progress has been slow. Multiple factors can influence clinical outcomes for patients with CKD, including but not limited to underlying predisposing medical conditions, genetic risks, environmental, sociocultural factors and others such as healthcare systems and access to healthcare [6–12]. These factors may also lead to disparities in incidence and prevalence across different patient subgroups while also limiting optimal care for all patients [6–12].

The Center for Kidney Disease Research, Education and Hope (CURE-CKD) registry was developed to capitalize on a unique opportunity to integrate and harmonize electronic health record (EHR) data on 9.9 million patients treated since 2006 within two large healthcare systems using key elements outlined by Goldstein et al. [13] and Navaneethan and colleagues [14]. The CURE-CKD registry is intended to provide unique insights into real-world clinical care and outcomes from a broad repository of over 3.3 million candidate patients with a single entry-point CKD criteria or at-risk for CKD, and a more select registry of over 2.6 million patients following stricter guide-line-based CKD or at-risk CKD criteria. The objectives of the CURE-CKD registry are to: 1) collaborate to develop standardized data structures for analysis and to harmonize two large and distinct datasets; 2) identify patients with CKD or at increased risk for CKD (hypertension (HTN), diabetes (DM), and prediabetes) from EHR data; 3) support site-combined and site-specific comparative analyses of key clinical issues including but not limited to, the prevalence of testing for CKD using laboratory measurements including estimated glomerular filtration rate (eGFR), urine albumin-to-creatinine ratio (UACR) and total urine protein-to-creatinine ratio (UPCR); the ability to examine eGFR decline to identify high-risk patients; the impact of evidence-based ambulatory care such as adherence to recommended pharmacotherapy, blood pressure and DM control on delaying eGFR decline and reducing rates of hospitalizations and re-hospitalizations; and 4) identify subgroups traditionally beset with disparities in CKD and at-risk for CKD outcomes (e.g. racial/ethnic minority, low income, rural dwelling/geolocation) and develop strategies to eliminate disparities in care. Given the origins of this data-rich registry, several unique analytic and decision-making methodologies were developed to produce a database representative of real-world data but also readily amenable to scientific inquiry. The goal of this report is to describe the CURE-CKD registry design and outline proposed analytic methods.

## Methods/design

### Ethical statement

Independent institutional review board (IRB) approvals were obtained from the Providence Saint Joseph Health (PSJH Health) and the University of California, Los Angeles Health (UCLA Health) healthcare systems (Providence IRB: 2043 CURE-CKD: CKD and At-Risk CKD Registry, and UCLA IRB: 15–001993 Assessing the Prevalence and Management of Chronic Kidney Disease). The data in the repository (single entry-point criteria for CKD or at-risk for CKD) and registry (strict CKD or at-risk CKD criteria) are maintained in accordance with the principles of the Declaration of Helsinki and local regulatory requirements. Informed consent was not required as this is a limited dataset and Health Insurance Portability and Accountability waivers are in place.

### Study design

PSJH and UCLA Health are not-for-profit healthcare systems that established a collaborative data use agreement to provide the framework for data sharing and stewardship in a secure electronic environment. PSJH operates 829 clinics and 50 hospitals in Washington, Oregon, Alaska, Montana, and California. UCLA Health has 170 clinics and 4 hospitals (with admitting privileges at > 15 hospitals) in Southern California. Each system uses the Epic EHR (Epic System Corporation), from which data for the repository and resulting registry were extracted. The larger repository and the CURE-CKD registry are updated annually to provide additional longitudinal data for existing participants, and to identify new participants who meet inclusion/exclusion criteria.

### Data validation and harmonization

Clinical (e.g. laboratory measurements, physical measurements, and medication records) and administrative encounter data were validated and harmonized between the PSJH and UCLA Health systems to create a single EHR-based dataset, suitable for analyses in this ongoing, observational, multicenter study. Data structures were proposed, discussed and approved by a multidisciplinary team of investigators at weekly meetings and twice-yearly in-person meetings over a two-year period. The disciplines represented in this collaboration included collaborators with expertise in clinical nephrology, endocrinology, general internal medicine, health services research, pharmaceutical sciences, health disparities, health economics, biostatistics, big database construction, machine learning and bioinformatics.

CURE-CKD data analysts harmonized and linked data structures, and clinicians conducted internal and inter-institutional validation of EHR laboratory values, medication records, and administrative encounter codes [14]. When discordance could not be resolved, the issues were brought to CURE-CKD weekly team meetings for discussion and resolution. UCLA Health and PSJH Health datasets were fully harmonized and maintained as both merged and independent datasets.

### Participants and inclusion/exclusion criteria

**Fig. 1:**
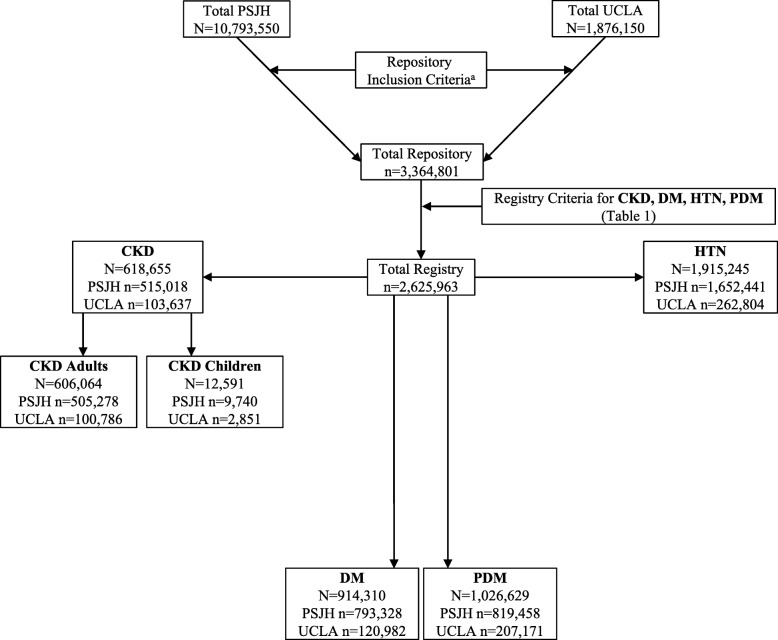
STROBE Diagram: Overview of participant groups by CKD and at-risk CKD categories in the Center for Kidney Disease Research, Education and Hope (CURE-CKD) Repository and Registry

Repository participants (*N* = 3,364,801) were identified from EHR laboratory and physical measurements, administrative codes and medication records from inpatient, outpatient and ambulatory settings (Fig. 1). Repository individuals were identified by a series of criteria including laboratory tests, with any eGFR (CKD-EPI) measurement <60 mL/min/1.73m^2^; any UACR ≥30 mg/g; any UPCR ≥150 mg/g; any hemoglobin A1c (HbA1c) ≥5.7%; any random blood glucose ≥140 mg/dL; or fasting blood glucose ≥100 mg/dL. Individuals with any diagnostic code for CKD, HTN, DM and prediabetes were identified as repository participants. Extracted physical measurements identified participants as repository eligible with any systolic or diastolic blood pressure ≥140 mm Hg or ≥90 mm Hg, respectively. Finally, medication records were examined, with individuals prescribed anti-hyperglycemic agents identified as repository participants.

Repository patients were then screened for inclusion in the CURE-CKD registry, following clinical practice guidelines (Table 1). Individuals with laboratory evidence of CKD (two measurements of eGFR <60 mL/min/1.73m^2^, UACR ≥30 mg/g, or UPCR ≥150 mg/g at least 90 days apart), or any encounter with a diagnostic code for CKD were entered into the registry (*N* = 618,655). For adults eGFR was calculated using the Chronic Kidney Disease Epidemiology (CKD-EPI) equation (15, 16) and for children with CKD (< 18 years) we use the bedside Schwartz equation [17]. Individuals with physical evidence (two measurements of systolic or diastolic blood pressure ≥ 140 mm Hg or ≥90 mm Hg, respectively at least 14 days apart) or any encounter with a diagnostic code for HTN were entered into the registry (*N* = 1,915,245; please note that patients can have more than one disorder so the sample sizes are not mutually exclusive). Individuals were identified and entered into the registry (*N* = 91,310) if there was laboratory evidence of DM (one measurement of HbA1c ≥6.5%; two measurements of random or fasting blood glucose ≥200 mg/dL or ≥126 mg/dL, respectively at least 1 day, but not more than 2 years apart); one inpatient encounter or at least two outpatient encounters with a diagnostic code of DM; or at least one prescription for anti-hyperglycemic medication. Anti-hyperglycemic medications were identified by therapeutic classes (insulin, sulfonylurea, thiazolidinedione, dipeptidyl peptidase 4, glucagon-like peptide 1, sodium glucose co-transporter 2, and metformin). Anti-hypertensive, and nephrotoxic medications (nonsteroidal anti-inflammatory drugs (NSAIDs) and proton pump inhibitors (PPIs) were also identified. Individuals prescribed metformin with a diagnostic code indicating polycystic ovarian syndrome, with no other evidence of DM or prediabetes inclusion criteria were subsequently removed from the registry. All included medications were manually reviewed and verified by the study pharmacist and/or clinical team members. Individuals with laboratory evidence of prediabetes (one measurement of HbA1c between 5.7–6.4%; two measurements of random or fasting blood glucose between 140 and 199 mg/dL or 100–125 mg/dL, respectively at least 1 day, but not more than 2 years apart); or any encounter with a diagnostic code indicating prediabetes were entered into the registry (*N* = 1,026,629).

**Table 1:** Inclusion Criteria for the CURE-CKD Registry

Chronic Kidney Disease [15–17]	1. At least two eGFR (CKD-EPI equation or Schwartz) measurements <60 mL/min/1.73m^2^ at least 90 days apart, or 2. At least two laboratory measurements at least 90 days apart in which albuminuria was indicated; albumin to creatinine ratio ≥30 mg/g, or total urine protein ≥150 mg/g, or 3. At least one encounter (inpatient or outpatient) with an ICD-9 or ICD-10 diagnosis code indicating chronic kidney disease is present
Hypertension [18]	1. At least two vital sign measurements >14 days apart with a systolic blood pressure ≥140 mm Hg or diastolic blood pressure ≥90 mm Hg, or 2. At least one encounter (inpatient or outpatient) with an ICD-9 or ICD-10 diagnosis code indicating hypertension is present
Diabetes Mellitus [19, 20]	1. Any qualifying laboratory result: a. one HbA1c ≥6.5%, or b. two random blood glucose measurements ≥200 mg/dL, at least 1 day, but no more than 2 years apart, or c. two fasting blood glucose measurements ≥126 mg/dl, at least 1 day, but no more than 2 years apart, or 2. At least one medication record with a pharmaceutical class (i.e. anti-glycemic medications) for treating diabetes mellitus (excludes individuals diagnosed with PCOS taking metformin), or 3. At least two outpatient ICD-9 or ICD-10 diagnosis codes indicating diabetes mellitus is present, or 4. At least one inpatient ICD-9 or ICD-10 diagnosis code indicating diabetes mellitus is present
Pre-Diabetes Mellitus	1. Any qualifying laboratory result: a. one HbA1c 5.7–6.4%, or b. two random blood glucose measurements 140–199 mg/dL, at least 1 day, but no more than 2 years apart, or c. two fasting blood glucose measurements 100–125 mg/dl, at least 1 day, but not more than 2 years apart, or 2. At least one encounter (inpatient or outpatient) with an ICD-9 or ICD-10 diagnosis code indicating pre-diabetes mellitus

*eGFR* estimated glomerular filtration rate, *CKD* Chronic Kidney Disease, *ICD-9/ICD-10* International Classification of Diseases, 9th Revision/10th Revision, *Hb* hemoglobin, *PCOS* Polycystic ovarian syndrome, *CKD-EPI* Chronic Kidney Disease Epidemiology Collaboration

### Characterizing registry participants

Registry patients have been characterized by clinical and demographic characteristics [21]. Additionally, registry patients have been classified by geography, including state and urban versus rural status. A majority of patients in the registry (*N* = 2,625,963) currently reside in the states of Washington (41.6%), California (31.5%), Oregon (17.2%), Alaska (3.4%) and Montana (2.6%). Patient resident zip codes were mapped to Rural-Urban Commuting Area (RUCA) codes, following category C (https://depts.washington.edu/uwruca/ruca-uses.php). Registry patients have been classified as urban (87.5%) and rural (11.4%). Any individuals without a zip code (1.1%) were not assigned a RUCA code and were not classified as either living in a rural or an urban area.

### Planned CURE-CKD registry analyses

#### Outcomes assessment

The CURE-CKD study team will assess changes in laboratory and physical markers including eGFR, UACR/UPCR, and blood pressure, as well as adherence to effective and de-implementation of ineffective strategies/medicines over time in registry participants. Both kidney replacement therapy (hemodialysis, peritoneal dialysis, kidney transplant) and mortality will be obtained by linking the CURE-CKD registry to the United States Renal Data System (USRDS) Coordinating Center through a USRDS-merged dataset agreement for release of data with limited personally identifiable information. The USRDS provides data solely for the conduct of legitimate and approved biomedical, cost-effectiveness, and other economic research. To obtain accurate mortality data, the CURE-CKD registry will link to the National Death Index provided by the Centers for Disease Control and Prevention, the Social Security Death Master File, and to state death indices for the states served by PSJH Health and UCLA Health.

### Traditional statistical analyses

Planned statistical approaches include descriptive analyses of the dataset as combined and as two distinct health systems. Statistical modeling approaches such as linear regression, generalized estimating equations, and linear mixed models (LMMs) will be used to investigate change in eGFR over time. LMMs have been shown to be the most robust approach to address the varying number and dispersion of time points and differences in duration of follow-up, especially in settings with high drop-out rates (e.g. slope of eGFR decline accounting for initiation of kidney replacement therapy and death) [22]. Notably, such a framework also allows for the examination of non-linear patterns of change over time (e.g., quadratic change, piecewise change) and lends itself well to extensions of LMMs such as finite growth mixture modeling for the examination of population-level heterogeneity into distinct, empirically-driven sub-groups of meaningful change. LMMs will be used in multivariable models to examine differences in eGFR trajectories, change in UACR/UPCR, and other clinical parameters, controlling for baseline demographics, clinical comorbidities, location (using small area analyses with geocoded data) and time-varying covariates (systolic blood pressure, HbA1c, use of NSAIDs and angiotensin converting enzyme inhibitors (ACEI) or angiotensin II receptor blockers (ARB), both overall and in known disparate subgroups (e.g. age, race/ethnicity, gender, socioeconomic status, rurality). Time to event analysis (Kaplan-Meier and Cox proportional hazard regression models) will be used to examine CKD and at-risk CKD differences in rates reaching clinically significant declines in eGFR, ESKD and mortality, progression to incident DM and others. Competing risk analyses will be conducted when appropriate given both dialysis and kidney transplant compete with the outcome of death.

### Machine learning analyses

**Fig. 2:**
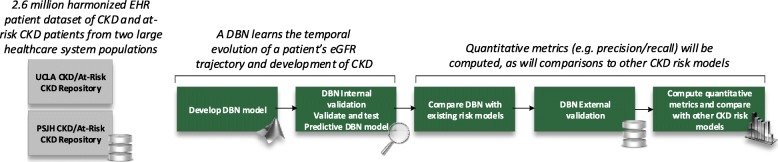
Summary of Dynamic Belief Network Model

In addition to traditional regression modeling of outcomes, machine learning methods will be used to construct dynamic belief networks (DBNs) to model changes in eGFR and to estimate the probability of developing advanced CKD over time. The DBNs will examine factors contributing to eGFR over time, and differences in eGFR trajectories between subgroups. The DBN’s predictive performance will be compared against existing validated CKD risk models [23–27] and other machine learning-based methods. In addition, the DBN-based models will be tested to determine if the models correctly predict changes in eGFR trajectory by assessing predictions at different time points relative to known outcomes. Internal validity of the DBN will be assessed by its capability to predict the change in eGFR trajectory based on past observations, and the external validity by cross-testing between differing sites (Fig. 2), with content expert review of transportability of findings across sites and to external populations. Model performance will be tested in terms of discrimination (assessing the model’s ability to distinguish among patients with different outcomes) and calibration (c-statistics, comparing observed and predicted event rates for groups of patients).

## Discussion

The CURE-CKD registry will provide new approaches to fill knowledge gaps and guide the development of better management strategies for patients with and at-risk for CKD. The large volume of data, with over 3.3 million unique patients available in the combined repository and over 2.6 million patients in the registry, offers an opportunity to conduct a myriad of health services-related studies (e.g. epidemiological research, machine learning, clinical decision support, team-based interventions, value-based care, reduction in health disparities) for this patient population and traditionally under-represented disadvantaged sub-populations (e.g., American Indian or Asian American populations, rural-dwelling populations) in diverse real-world settings [28].

The CURE-CKD study team will examine multiple aspects of evidence-based care that have not been extensively validated using real-world data. For instance, uncertainty persists regarding the appropriate blood pressure target levels for CKD patients [29, 30]. Some studies support a lower target blood pressure goal [31, 32], some have found no relationship between CKD-related outcomes and blood pressure [33], while others have found blood pressure-related outcomes vary by the severity of UACR/UPCR, CKD stage, presence of DM, and other factors [34–38]. These conflicting findings have led to consternation in the clinical care of CKD patients. The recent 2017 American Heart Association’s current blood pressure guideline recommends the goal blood pressure <130/80 mm Hg for all CKD patients without consideration of CKD stage [39]. Prospective studies using large real-world datasets such as the CURE-CKD registry, may better assist with informing evidence-based care for patients with and at-risk for CKD, including optimal targets for different patient subgroups (e.g. those with comorbid conditions, different CKD stages). The use of NSAIDs [40–42], PPIs [43–46], and smoking [47–49] have been reported to be associated with CKD onset, progression, and poor outcomes. The CURE-CKD registry is substantially powered to examine the association of these and other CKD risk factors in relation to several CKD protective or resilience factors stratified by patient subgroups. It is also well positioned to investigate the contributions of DM, prediabetes and/or HTN to CKD incidence, which will enable the development of clearer approaches to practice-based algorithms for promotion of early detection and intervention for CKD as well as more accurate prediction of disease progression.

In health disparate populations such as minority racial/ethnic groups, there is a paucity of data on the role of major modifiable risk factors such as protective and potentially harmful medications, smoking, body weight, and lifestyle in CKD-related outcomes in real-world settings. For instance, while prior studies found the degree of blood pressure lowering with ACEI or ARB use was greater in Whites than in Blacks [50] leading to low use of ACEI/ARBs in Blacks, the African American Study of Kidney Disease and Hypertension demonstrated that inhibition of the renin-angiotensin-aldosterone system was the most effective class of blood pressure therapy in improving CKD and mortality outcomes in Blacks with hypertension-related CKD [51], and has led to an improvement in the practice of low ACEI/ARB use in Blacks. Further exploration of ACEI/ARBs in clinical outcomes for Blacks and other racial/ethnic groups in clinical practice is warranted. The CURE-CKD registry provides a large, real-world longitudinal dataset to evaluate conflicting results from trials with observations in a clinical practice setting. Developing a better understanding of key modifiable risk factors and their interaction with existing clinical targets could lead to new anti-hypertensive medication recommendations for select subgroups of patients with CKD and especially for those patients with rapid progression of eGFR decline.

The collaborative nature of the CURE-CKD registry has inherent barriers that must be overcome in the development of inter-institutional EHR-based registries. In general terms, these limitations may include data quality, data inconsistency or stability (e.g. lack of data standards, variations across laboratories), the validation of data and other analytical limitations (e.g. missing data, potential over-fitting of prediction models, multiple comparisons, risk of false-positive associations), trust building and the development of data use agreements that protect all collaborative institutions and the inherent limitations of observational data [52]. More specific limitations include differences in documentation practices that exist across and between healthcare systems [53] even with a similar EHR platform. Additional limitations for inter-institutional registries such as the CURE-CKD registry includes attrition rates that may vary regionally due to insurance coverage, rates of poverty, implementation or de-implementation of the Affordable Care Act and other state or national healthcare initiatives.

By contrast, the CURE-CKD registry has many strengths. These include a two-year preparation period to create a robust inter-institutional registry using close and thoughtful collaboration to define common structures and to identify and synchronize data elements. The initial iteration of the registry includes longitudinal data over an 11-year period, from 2006 to 2017, with annual updates. Another strength is the use of laboratory and clinical data including disease-specific (e.g. DM, prediabetes) medications to supplement administrative encounter data, rigorous data curation and longitudinal observation of a large number of registry participants. For longitudinal assessment of major clinical outcomes, the CURE-CKD registry will be linked with national and state death indices and the USRDS to ascertain ESKD events and Medicare administrative data for hospitalization events. Also, the proportion of patients with HTN (73%) and DM (31%) in the registry are similar to the participants in the Kidney Early Evaluation Program [54, 55] providing a level of external validation. To address the low use of administrative encounter codes especially for conditions such as CKD, HTN, DM, prediabetes, and other comorbidities, CURE-CKD inclusion criteria consisted of clinical and laboratory data as well as medication records. Finally, it is important to note that real-world observations from EHRs can be used to supplement randomized trials to inform best practices and clinical guidelines as well as to generate CKD- and at-risk for CKD-based interventions. In the future, this longitudinal data source combined with statistical methods such as propensity score matching that identify robust comparator groups, will be an efficient learning lab to study the impact of real world system level interventions designed to prevent the onset of CKD in high risk populations and to reduce the rate of persons with rapid eGFR decline among those with CKD. To conduct research that can improve health equity, it will never be possible to conduct randomized controlled trials in all the groups at greatest risk, so approaches that use robust real-world data systems with unbiased comparator groups such as CURE-CKD hold promise for identifying the interventions that reduce disparities the most.

In summary, the development of novel methods to improve the identification and early intervention for patients with or at-risk for CKD has remained a challenge [56]. Big data analytics from EHRs have tremendous potential to improve the quality and outcomes of care for patients with and at-risk for CKD. With the emerging addition of social determinants of health and precision medicine (i.e. omics) markers to patients in large healthcare systems, the amount of data available to inform CKD care and research will soon be exponential in nature. A combination of traditional and machine learning-based analytic approaches will be critical to appropriately analyze these rapidly growing datasets with careful interpretation to retain their relevance for patient care, clinical management, and performance improvement. The CURE-CKD registry not only includes comprehensive administrative encounter data, but also includes a vast amount of clinical and laboratory measurements, as well as pharmacy and procedure records. The CURE-CKD study team is well positioned to conduct robust longitudinal analyses that will include important subgroups, with much greater power than most existing sources to identify subgroup-level differences. CURE-CKD has the potential to provide important contributions for healthcare in patients with and at-risk for CKD using observations from real-world settings and to provide timely opportunity to respond to the recent Executive Order on Advancing American Kidney Health [57].

## Data Availability

N/A.

## Abbreviations

ACEI: Angiotensin Converting Enzyme Inhibitor
ARB: Angiotensin II Receptor Blockers
CKD: Chronic Kidney Disease
CURE-CKD: Center for Kidney Disease Research, Education and Hope
DBNs: Dynamic Belief Networks
DM: Diabetes Mellitus
eGFR: Estimated Glomerular Filtration Rate
EHR: Electronic Health Record
ESKD: End-Stage Kidney Disease
HbA1c: Hemoglobin A1c
HTN: Hypertension
IRB: Institutional Review Board
LMMs: Linear Mixed Models
NSAIDs: Nonsteroidal Anti-Inflammatory Drugs
PPIs: Proton Pump Inhibitors
PSJH Health: Providence Saint Joseph Health
RUCA: Rural-Urban Commuting Area
UACR: Urine Albumin-to-Creatinine Ratio
UCLA Health: University of California, Los Angeles Health
UPCR: Urine Protein-to-Creatinine Ratio
US: United States
USRDS: United States Renal Data System
